# Changing Expression Profiles of Messenger RNA, MicroRNA, Long Non-coding RNA, and Circular RNA Reveal the Key Regulators and Interaction Networks of Competing Endogenous RNA in Pulmonary Fibrosis

**DOI:** 10.3389/fgene.2020.558095

**Published:** 2020-09-24

**Authors:** Xue Liu, Huaman Liu, Xinhua Jia, Rong He, Xinyue Zhang, Wei Zhang

**Affiliations:** Department of Respiration, Affiliated Hospital of Shandong University of Traditional Chinese Medicine, Jinan, China

**Keywords:** pulmonary fibrosis, whole transcriptome sequencing, ceRNA, co-expression network, regulation

## Abstract

Pulmonary fibrosis is a kind of interstitial lung disease with architectural remodeling of tissues and excessive matrix deposition. Apart from messenger RNA (mRNA), microRNA (miRNA), long non-coding RNA (lncRNA), and circular RNA (circRNA) could also play important roles in the regulatory processes of occurrence and progression of pulmonary fibrosis. In the present study, the pulmonary fibrosis model was administered with bleomycin. Whole transcriptome sequencing analysis was applied to investigate the expression profiles of mRNAs, lncRNAs, circRNAs, and miRNAs. After comparing bleomycin-induced pulmonary fibrosis model lung samples and controls, 286 lncRNAs, 192 mRNAs, 605 circRNAs, and 32 miRNAs were found to be differentially expressed. Gene Ontology (GO) and Kyoto Encyclopedia of Genes and Genomes (KEGG) analyses were performed to investigate the potential functions of these differentially expressed (DE) mRNAs and non-coding RNAs (ncRNAs). The terms related to inflammatory response and tumor necrosis factor (TNF) signaling pathway were enriched, implying potential roles in regulatory process. In addition, two co-expression networks were also constructed to understand the internal regulating relationships of these mRNAs and ncRNAs. Our study provides a systematic perspective on the potential functions of these DE mRNAs and ncRNAs during PF process and could help pave the way for effective therapeutics for this devastating and complex disease.

## Introduction

Pulmonary fibrosis is a kind of lung disease that is a progressive and life-threatening pathologic process resulting in organ failure. This fibroproliferative disease resulted from a various group of lung disorders with similar clinical, pathophysiologic, and radiographic characteristics and are known as interstitial lung disease (Pardo and Selman, 2002). Pulmonary fibrosis developed after many factors, not only injuries but also viral infections, exposure to radiotherapy, chemotherapeutic drugs, and aerosolized environmental toxins (Denham and Hauer-Jensen, 2002; Kelly et al., 2002; Fubini and Hubbard, 2003; Chen and Stubbe, 2005). During the pulmonary fibrosis process, there are various physiological processes involved including severe scarring of the respiratory membrane, abnormal tissue repair, damage of lung tissue, fibroproliferation, and deposition of extracellular matrix. Fibrotic tissue replaced normal lung parenchyma and finally caused loss of pulmonary function (Crystal et al., 2002). It has been reported that plenty of pathways were involved in the pathogenesis of pulmonary fibrosis, such as inflammation, proteolytic/antiproteolytic imbalance, coagulation, angiogenesis, and apoptosis (Antoniou et al., 2007). The wound-healing response of pulmonary fibrotic conditions can be classified into three distinct phases: injury, inflammation, and repair (Wilson and Wynn, 2009). In addition, many key genes, such as transforming growth factor beta (TGF-β), interleukin (IL) gene family members, vascular endothelial growth factor (VEGF), platelet-derived growth factor (PDGF), are involved in the wound-healing response of pulmonary fibrotic condition (Minshall et al., 1997; Elovic et al., 1998; Ando et al., 2010).

In organisms, RNAs are described as an intricate interplay among diverse RNA, including protein-coding messenger RNAs (mRNAs) and non-coding RNAs (ncRNAs), microRNA (miRNAs), pseudogenes, and circular RNAs (circRNAs). These RNA transcripts act as competing endogenous RNAs (ceRNAs) and are important post-transcriptional regulators of gene expression (Salmena et al., 2011). As a kind of non-coding RNA, miRNA serves as a critical regulator in gene expression and is involved in a variety of crucial developmental, physiological, and disease processes such as pulmonary fibrosis (Ambros, 2001; He and Hannon, 2004). For instance, miR-326 is reported to inhibit TGF-β expression and attenuate the fibrotic response. In addition, more profibrotic genes such as smad3, est1, and MMP-9 could also be down-regulated by miR-326 (Das et al., 2014). MiR-21 regulates the activation of lung fibroblasts, while miR-155 targets keratinocyte growth factor to regulate the process (Pottier et al., 2009; Liu et al., 2010). Besides, miR-126 is involved in cystic fibrosis by regulating the expression profiles of TOM1 (Oglesby et al., 2010).

Long ncRNAs (lncRNAs) could also participate into the regulatory process of pulmonary fibrosis. For instance, up-regulated lncRNA-CHER could derepress the inhibition of miR-489 on MyD88 and Smad3 and then activates inflammation and fibrotic signaling pathway (Wu et al., 2016). LncRNA-MALAT1 serves as a target of miR-503 and triggers the downstream fibrotic signaling pathway, such as PI3K/AKT/mTOR pathways in silica-induced pulmonary fibrosis (Yan et al., 2017). According to the previous studies, lncRNA H_19_ promotes lung fibrosis through lncRNA H_19_-miR-197a and lncRNA H_19_-miR-29b interactions (Tang et al., 2016; Lu et al., 2018). As another kind of ceRNA, circRNAs also participated in the regulatory processes of pulmonary fibrosis. circHIPK2 promotes the activation of astrocyte and fibroblast through up-regulation of the expression of sigmar1 (Cao et al., 2017; Huang et al., 2017). Besides, circHECTD1 has been proved to promote the silica-induced pulmonary fibrosis via HECTD1 (Fang et al., 2018). In addition, silica-induced initiation of circZC3H4 RNA/ZC3H4 pathway promotes the pulmonary macrophage activation (Yang et al., 2018). With the development of sequencing technology, whole transcriptome sequencing analyses had been widely applied to investigate the interactions between miRNAs–lncRNAs–circRNAs–mRNAs in various physiological processes, such as bronchopulmonary dysplasia, osteoclastogenesis, and bladder cancer (Dou et al., 2016; Li et al., 2018; Wang et al., 2019).

In this study, expression profiles of mRNAs, lncRNAs, miRNAs, and circRNAs were obtained through whole transcriptome sequencing analyses. Then, a comparison between bleomycin-induced pulmonary fibrosis model lung samples and controls was applied to screen the differentially expressed (DE) mRNAs and ncRNAs, which were the potential key regulators in the pulmonary fibrosis process. Gene Ontology (GO) and Kyoto Encyclopedia of Genes and Genomes (KEGG) enrichment analysis were performed to simplify the filtration process and investigate the involved biological process (BP) and KEGG signal pathway. Finally, a set of potential mRNAs and ncRNAs involved in the fibrotic process were selected; lncRNA–miRNA–mRNA and circRNA–miRNA–mRNA co-expression networks were constructed to exhibit the potential ceRNA regulatory relationships in co-expression network. The findings facilitate our understanding of regulatory mechanisms, supply fundamental support for further research in pulmonary fibrosis, and lead to new theories for the pathogenesis and treatment of pulmonary fibrosis.

## Materials and Methods

### Ethics Approval and Consent to Participate

All the experiments were conducted according to the Guidelines for the Institutional Animal Care and Use Committee of Affiliated Hospital of Shandong University of Traditional Chinese Medicine.

### Bleomycin-Induced Pulmonary Fibrosis Model

Twenty specific path-free Sprague–Dawley (SD) rats of 7 weeks old were raised in the laboratory. Then they were randomly divided into two groups, the pulmonary fibrosis model group and the control group. The model ones were administered with 7 mg/kg of bleomycin (Sigma, United States) through a single intratracheal instillation under anesthesia, while the control rats were administered with equal volume of saline (Szapiel et al., 1979). After 28 days of treatment, rats were killed, and lung specimens were collected for further analyses. Specimens were immediately frozen in liquid nitrogen and stored at −80°C for RNA extraction. All the experiment procedures were conducted in accordance with institutional animal and use committee of the Shandong University of Traditional Chinese Medicine.

### Masson Staining

Lung specimens were fixed with 4% paraformaldehyde overnight. After dehydrating in a series of ethanol and clearing in xylene, lung tissues were embedded in paraffin and cut into 4-μm sections. The sections were stained with Masson bluing solution and were examined under microscope to detect the severity of fibrosis.

### RNA Isolation and cDNA Library Construction and Illumina Sequencing

Total RNA was extracted from each sample with TRIzol reagent according to the manufacturer’s protocol (Invitrogen, Carlsbad, CA, United States). The quality and purity of total RNA were examined by 1.5% agarose gel electrophoresis, NanoPhotometer Pearl (Implen GmbH, Munich, Germany), and Agilent 2100 bioanalyzer (Agilent Technologies, Santa Clara, CA, United States). Approximately 1 μg of total RNA was applied to prepare small RNA library with TruSeq Small RNA Sample Prep Kits (Illumina, San Diego, United States) in accordance with the protocol. Then six libraries (three PF model rat and three control ones) were sequenced by Illumina Hiseq 2500, and 50 bp single-end reads were generated at LC-Bio (Hangzhou, China).

Another six cDNA libraries (three PF model rat and three control ones) were constructed to detect the expression profiles of mRNAs, lncRNAs, and circRNAs. Approximately 10 μg total RNA was used to deplete ribosomal RNA (rRNA) with Epicenter Ribo-Zero Gold Kit (Illumina, San Diego, United States). Following purification, the remaining RNAs were fragmented into small pieces using divalent cations under elevated temperature. The final cDNA libraries were reverse-transcribed from the cleaved RNA fragments with the mRNA-seq sample preparation kit (Illumina, San Diego, United States). Then, the libraries were subjected to paired-end sequencing of 150 bp on the Illumina Hiseq 4000 at LC-Bio (Hangzhou, China).

### Discovery of the Messenger RNAs, Long Non-coding RNAs, Circular RNAs, and MicroRNAs

The raw reads contained raw reads, low-quality bases, and undetermined bases were removed by Cutadapt (Martin, 2011). Then the sequence quality of remaining reads was validated by FastQC^1^. Clean reads were mapped to the reference genome of rat, *Rattus norvegicus*, by Bowtie2 and Tophat2 with the default parameters (Langmead and Salzberg, 2012; Kim et al., 2013). Then StringTie was applied to assemble the mapped reads of each sequencing libraries (Pertea et al., 2015), and a comprehensive transcriptome was reconstructed through merging all the samples. StringTie and Ballgown were used to estimate the expression profiles of all the transcripts (Frazee et al., 2015).

Transcripts that overlapped with known mRNAs and shorter than 200 bp were filtered. Coding potentials of these transcripts were predicted by CPC, CNCI, and Pfam (Kong et al., 2007; Punta et al., 2011; Sun et al., 2013). According to the prediction results, the transcript with CPC score < −1 and CNCI score < 0 were discarded. The remaining transcripts with class code (l,j,o,u, and x) were considered as lncRNAs.

According to the pipeline used in the analysis of mRNA discovery, unmapped reads were still mapped to the genome by Tophat-fusion (Kim and Salzberg, 2011). CIRCExplorer was used to *de novo* assemble the mapped reads to circRNAs (Zhang et al., 2014, 2016). Then back splicing reads were identified in unmapped reads through tophat-fusion and CIRECExplorer. Then the expression profiles for circRNAs were calculated by in-house scripts.

Raw reads generated from microRNA sequencing libraries were subjected to ACGT101-miR (LC Sciences, Huston, TX, United States) to filter out adapter dimers, junk, low complexity, repeats, rRNA, transfer RNA (tRNA), small nuclear RNA (snRNA), and small nucleolar RNA (snoRNA). Unique sequences varying from 18 to 26 nucleotides were mapped to rat precursors in miRbase 21.0 by BLAST in order to identify known miRNAs and novel 3p- and 5p-derived miRNAs. The unique sequence mapping to the rat mature miRNAs in hairpin arms was considered to be known miRNAs. The unique sequence mapping to the other arm of known rat precursor hairpin opposite to the annotated mature miRNA containing arm was identified as miRNAs. The remaining sequences were mapped to other selected species precursors; then they were further mapped to rat genome to determine the genomic locations. They were also defined as known miRNAs. The unmapped sequences were applied to predict novel miRNAs through RNAfold^2^.

### Differential Expression Analysis

Expression profiles of mRNAs and lncRNAs were calculated by StringTie and R package Ballgown through FPKM (Frazee et al., 2015; Pertea et al., 2015). mRNAs and lncRNAs with *p* ≤ 0.05 and |log2foldchange| ≥ 1 were identified as differentially expressed. DESeq v1.16.0 was used for differential expression analysis between PF model and control replicates by a model based on the negative binomial distribution. CircRNAs with *p* ≤ 0.05 were regarded as DE ones. MiRNAs with *p* ≤ 0.05 and |log2foldchange| ≥ 1 were identified as DE miRNAs. Multiple testing correction has been performed during statistical analysis, and FDR and *q-*value were used to adjust the results of DE RNAs.

### The Prediction of Target Genes of Long Non-coding RNAs and MicroRNAs

LncRNAs may play a cis role on neighboring target genes. In this study, coding genes distributed in the 100,000 bp upstream and downstream regions were filtrated out and considered as target genes of lncRNAs. Targets of miRNAs were predicted to construct the lncRNA/circRNA–miRNA–mRNA networks. miRanda 3.3a and TargetScan were applied to identify miRNA binding sites in lncRNAs, circRNAs, and mRNAs (John et al., 2004; Lewis et al., 2005). Then, the predicted results through these two algorithms were combined, and the intersection was calculated.

### Gene Ontology and Kyoto Encyclopedia of Genes and Genomes Pathway Analysis

In order to filter the potential mRNAs and ncRNAs playing important roles in rat PF, GO and KEGG enrichment analyses were carried out. The terms with *p* ≤ 0.05 were indicated to be significantly enriched GO and KEGG terms.

### qRT-PCR Validation

According to the DE RNAs, ceRNAs were selected to validate the expression profiles in control group and model group by qRT-PCR. The RNA used for Illumina sequencing was also used here for the validation. Roche LightCycler 480 (Roche, Forrentrasse, Switzerland) was applied to perform qRT-PCR with SYBR Premix Ex Taq II (TaKaRa, Dalian, China). DAPDH and U6 were used as the reference genes to normalize the relative expression. Primers used in the research are shown in Supplementary Table S1. Gene relative expression level was analyzed by 2^–Δ^ ^Δ^ ^Ct^ method. Statistical analysis was performed by SPSS 20.0. Differences with *p* ≤ 0.05 were considered significant.

## Results

### Validation of Pulmonary Fibrosis Animal Model

**FIGURE 1:**
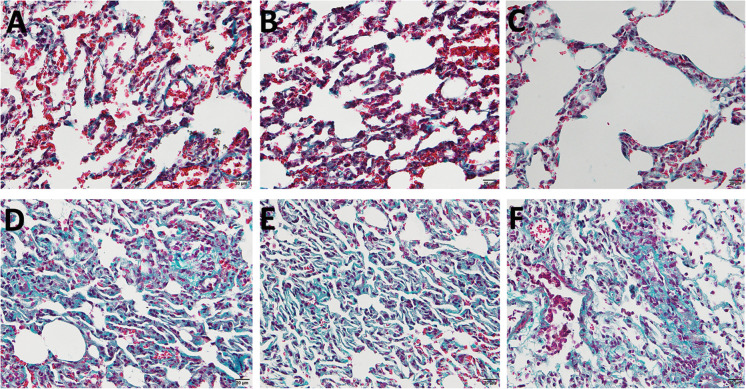
The Masson sections of Sprague–Dawley (SD) rat pulmonary. **(A–C)** The control group treated with saline. **(D–F)** The model group treated with bleomycin. Blue area represents fibrous nodules.

Masson staining was applied to identify whether the pulmonary fibrosis rat model was successfully established. As is shown in Figure 1, the alveolar structures in control lung specimens were complete and continuous without obvious abnormality. Besides, the alveolar septum was thinner and contained little collagen fibers. On the contrary, a large number of lung fibrous nodules (blue area) that exist in the lung interstitium were observed in model group. All the features mentioned above indicated that pulmonary fibrosis rat model had been successfully established.

### Expression Profiles of Long Non-coding RNAs and Messenger RNAs in PF Models

**FIGURE 2:**
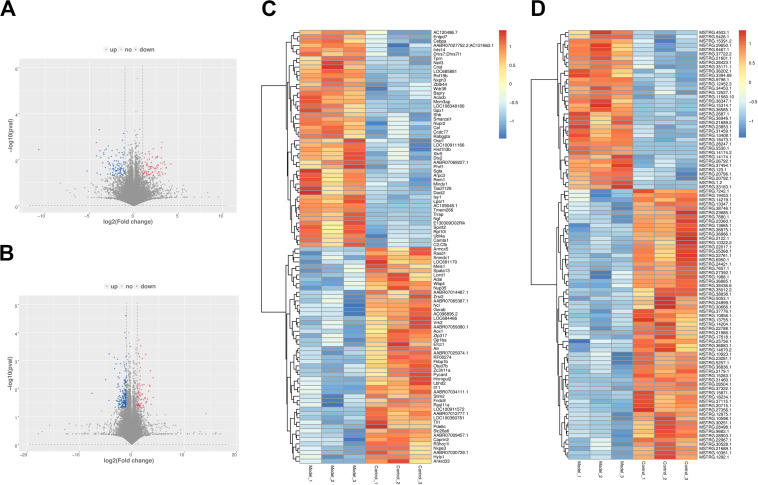
The expression profiles of long non-coding RNAs (lncRNAs) and messenger RNAs (mRNAs) in PF models. **(A)** Volcano plot of differentially expressed (DE) mRNAs between control group and model group. **(B)** Volcano plot of DE lncRNAs between control group and model group. Red dots represent the mRNAs or lncRNAs up-regulated in PF model samples, blue dots the down-regulated mRNAs or lncRNAs, and the gray dots the ones that showed no differences. **(C)** Heatmap of DE mRNAs between control group and model group. **(D)** Heatmap of DE lncRNAs between control group and model group.

A total of six cDNA libraries and six miRNA libraries were sequenced on the Illumina platform, encompassing about 35 Gb of sequence. The data have been uploaded to National Center for Biotechnology Information (NCBI) Sequence Read Archive (SRA) site, with accession numbers of GSE153296. Valid ratio of each library is shown in Tables 1, 2. As a result of this analysis, 43,627 genes and 19,844 lncRNAs were identified in all samples (Supplementary Tables S2, S3). Among these, 286 lncRNAs (including 87 up-regulated and 199 down-regulated) and 192 mRNAs (including 86 up-regulated and 106 down-regulated) were differentially expressed between PF model samples and control samples with *p* < 0.05 and | log2foldchange| ≥ 1 (Figure 2). Clear differences of lncRNAs and mRNAs between PF model samples and control ones are shown in the volcano plot (Figures 2A,B). In addition to this, a heatmap of hierarchical clustering of DE genes or lncRNAs was generated to visualize the overall pattern of gene expression (Figures 2C,D). All the DE mRNAs and lncRNAs are shown in Supplementary Tables S4, S5.

**TABLE 1:** Summary statistics of sequencing data.

Sample	Raw reads	Clean reads	Q20	Q30	GC
Control_1	101469372	92707318	99.88	97.23	48.50
Control_2	99057016	87434102	99.78	96.82	49.50
Control_3	102536772	96788582	99.93	97.64	47.50
Model_1	104833184	95584308	99.88	97.33	48.50
Model_2	98225620	88734774	99.85	97.25	49.00
Model_3	96740918	88936996	99.87	97.23	49.50

**TABLE 2:** Summary statistics of miRNA sequencing data.

Sample	Raw reads	Clean reads	Unique reads	Q30
Control_1_miRNA	15291456	10903843	761264	96.23
Control_2_miRNA	13801948	10022818	845695	95.92
Control_3_miRNA	11956735	8370568	893128	96.63
Model_1_miRNA	14355647	12017240	993372	96.71
Model_2_miRNA	12423822	10081951	889774	96.85
Model_3_miRNA	16478132	14025974	1033740	95.33

### Expression Profiles of Circular RNAs and MicroRNAs in PF Models

**FIGURE 3:**
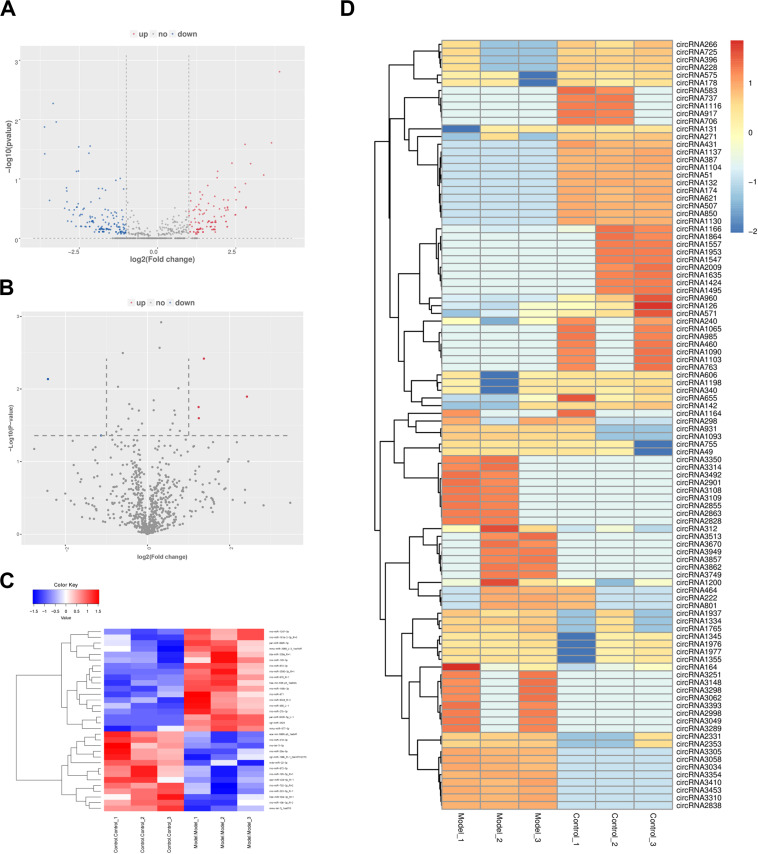
The expression profiles of circular RNAs (circRNAs) and microRNAs (miRNAs) in PF models. **(A)** Volcano plot of differentially expressed (DE) circRNAs between control group and model group. **(B)** Volcano plot of DE miRNAs between control group and model group. Red dots represent the circRNAs or miRNAs up-regulated in PF model samples, blue dots down-regulated circRNAs or miRNAs, and the gray dots the ones that showed no differences. **(C)** Heatmap of DE miRNAs between control group and model group. **(D)** Heatmap of DE circRNAs between control group and model group.

After prediction and filtration, 5,216 circRNAs and 798 miRNAs were identified in PF model and control samples. According to the comparison between model and control specimens, 605 circRNAs with | log2foldchange| ≥ 1 (including 287 up-regulated and 318 down-regulated) and 32 miRNAs with *p* < 0.05 and | log2foldchange| ≥ 1 (including 18 up-regulated and 14 down-regulated) were differentially expressed (Supplementary Tables S6, S7). In the volcano plot, the red plot represents the circRNAs or miRNAs up-regulated in PF model lungs, while the blue dots represent the down-regulated circRNAs or miRNAs (Figures 3A,B). A heatmap was constructed to obtain a hierarchical clustering of DE circRNAs and miRNAs. Different colors represent the different expression levels of the circRNAs and miRNAs (Figures 3C,D).

### Gene Ontology and Kyoto Encyclopedia of Genes and Genomes Enrichment Analysis of Messenger RNA, Long Non-coding RNA, Circular RNA, and MicroRNA

GO and KEGG pathway analyses were performed to filter the key regulators and pathways playing vital roles in the process of pulmonary fibrosis with DE mRNAs (DE mRNAs), *cis*/*trans*-regulated target genes of DE lncRNAs, hosting genes of DE circRNAs, and target genes of DE miRNAs. All genes were classified into three categories, including BP, cellular components (CCs), and molecular functions (MFs). Key regulators were obtained by terms in the BP category. The top 15 enriched GO terms and KEGG signal pathways were listed in scatter plots, which may be associated with mechanisms of pulmonary fibrosis. In the scatter plots, the size of the dots represents the gene numbers enriched in the GO terms. Besides, the *p* value of every enriched term was represented by different colors (Supplementary Figure S1).

**FIGURE 4:**
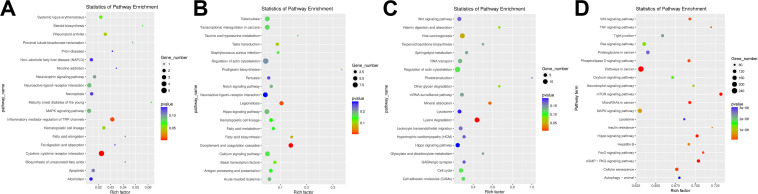
Kyoto Encyclopedia of Genes and Genomes (KEGG) pathway analysis enrichment of differentially expressed (DE) competing endogenous RNAs (ceRNAs). **(A)** KEGG pathway analysis enrichment of DE mRNAs. **(B)** KEGG pathway analysis enrichment of DE long non-coding RNAs (lncRNAs). **(C)** KEGG pathway analysis enrichment of DE circRNAs. **(D)** KEGG pathway analysis enrichment of DE miRNAs.

In the GO enrichment results of DE mRNA GO (Supplementary Figure S1A), it showed that many potential pulmonary fibrosis-related terms were significantly enriched such as “lymphocyte homeostasis,” “positive regulation of IL-1 secretion,” “regulation of signaling receptor activity,” and “regulation of neuron differentiation” (Supplementary Table S8). It indicated that the immune system, neuron system, and various signaling transduction pathways had played important roles in the process of pulmonary fibrosis. Besides, many pulmonary fibrosis-related pathways were also enriched through KEGG analysis, such as “Cytokine-cytokine receptor interaction,” “inflammatory mediator regulation of TRP channel,” and “apoptosis” (Figure 4A and Supplementary Table S8).

According to the GO enrichment results of DE lncRNAs (Supplementary Figure S1B), we can find that many potential GO terms were associated with the pulmonary process, such as “cardiac muscle cell apoptotic process,” “neuronal action potential,” and “actin filament reorganization” (Supplementary Table S9). In addition, “Notch signaling pathway” and “regulation of actin cytoskeleton” KEGG pathway indicated that DE may regulate the pulmonary process in the aspect of apoptosis, nervous system, and cytoskeleton system (Figure 4B and Supplementary Table S9).

As shown in Supplementary Figure S1C, many PF-related GO terms were enriched in DE circRNA, such as “negative regulation of epidermal growth factor,” “negative regulation of ERK1 and ERK2 cascade,” and “positive regulation of TGF-β receptor signaling pathway” (Supplementary Table S10). Apart from these, various KEGG pathways including “lysine degradation,” “regulation of actin cytoskeleton,” “cell adhesin molecules (CAMs),” and “Wnt signaling pathway” were inferred to be related to the regulation process of PF (Figure 4C and Supplementary Table S10).

In the top 15 GO terms of DE miRNAs, we can find many related terms such as “positive regulation of apoptotic process.” Apart from these, many insignificantly enriched GO terms were also associated with the pulmonary fibrosis process including “angiogenesis” and “Wnt signaling pathway” (Supplementary Figure S1D and Supplementary Table S11). In the KEGG pathway enrichment analysis, “Wnt signaling pathway,” “tumor necrosis factor (TNF) signaling pathway,” “MAPK signaling pathway,” and “neurotrophin signaling pathway” were significantly enriched (Figure 4D and Supplementary Table S11).

### Construction of the Long Non-coding RNA–Messenger RNA Co-expression Network

**FIGURE 5:**
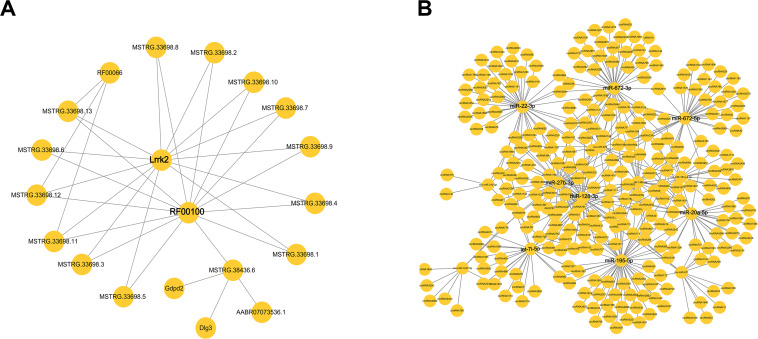
The network of differentially expressed (DE) competing endogenous RNAs (ceRNAs). **(A)** The interaction of microRNAs (mRNAs) and long non-coding RNAs (lncRNAs). **(B)** The interaction of cirRNAs and miRNA.

An lncRNA–mRNA co-expression network was constructed to show a complex interaction between lncRNAs and mRNAs. One lncRNA could regulate more than one gene in different ways, while one gene could also be regulated by multiple lncRNAs. As shown in Figure 5A, the DE lncRNAs and mRNA were used to construct the co-expression network. The result showed that Lrrk2 and RF00100 had the most interaction relationship with lncRNAs.

### Construction of the Circular RNA–MicroRNA Co-expression Network

Another circRNA–miRNA co-expression network was constructed to show a complex interaction between circRNAs and miRNAs. CircRNAs played important roles as a sponge to regulate miRNA, while one miRNA could also target more than one circRNA. As shown in Figure 5B, miR-22-3p, miRNA-672-3p/5p, miR-20a-5p, miR-195-5p, let-7i-5p, miR-27b-3p, and miR-128-3p had the most interaction with circRNAs. There miRNAs might have the function involved in pulmonary process.

### qRT-PCR Validation

**FIGURE 6:**
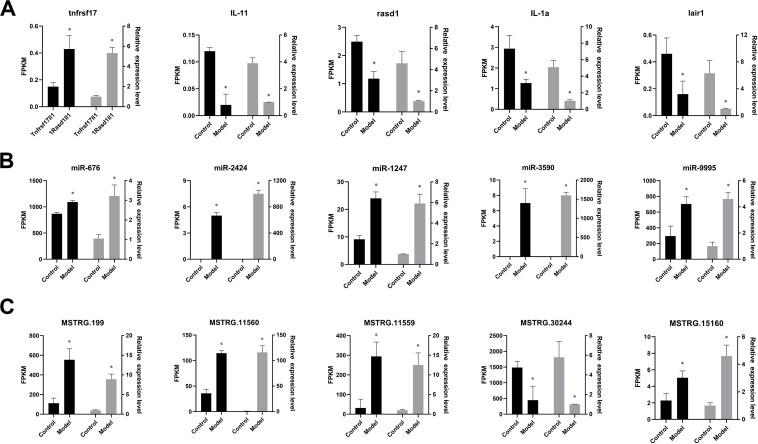
Verification of the expression patterns in both qRT-PCR and RNA-seq. The data are shown as mean ± SD (*n* = 3). Groups with different asterisks are significantly different (*p* < 0.05). **(A)** The expression pattern of mRNA in both qRT-PCR and RNA-seq. **(B)** The expression pattern of miRNA in both qRT-PCR and RNA-seq. **(C)** The expression pattern of lncRNA in both qRT-PCR and RNA-seq.

A set of ceRNAs of differential expression was validated by qRT-PCR including five mRNAs (tnfrsf17, IL-11, rasd1, IL-1a, and lair1), five miRNAs (miR-676, miR-2424, miR-1247-5p, miR-3590-3p, and miR-9995-3p), and five lncRNA (MSTRG.199, MSTRG.11560, MSTRG.11559, MSTRG.30244, and MSTRG.15160). Expression profiles of these selected ceRNAs are shown in Figure 6. It displays a similar expression pattern between sequencing data and qRT-PCR data.

## Discussion

Pulmonary fibrosis, a chronic fibrosing interstitial lung disease, is of unknown etiology and currently untreatable. It is imperative that detailed and integrated understanding of the cellular and molecular mechanisms of pulmonary fibrosis should be focused on. And these are helpful for the effective therapeutics for the disease. In this study, we applied high-throughput sequencing technology to investigate the potential regulatory mechanisms in the process of pulmonary fibrosis through DE mRNAs and ncRNAs.

It was found that many related genes and terms were enriched, including immune-related terms, nervous system-related terms, and epithelial cell development. In this study, nerve growth factor (NGF) and IL were observed, and it was speculated that they might be related with pulmonary fibrosis. It was reported that NGF plays important roles in the nervous system, endocrine system, and immune system (Levi-Montalcini, 1987; Aloe et al., 1997). In addition, NGF also participates in inflammatory responses and regulates the fibroblast migration (Micera et al., 2001). Besides, IL-1a and IL-11 were also significantly enriched in many immune-related GO terms, such as “inflammatory response,” “connective tissue replacement involved in inflammatory response wound healing,” and “positive regulation of IL-2 biosynthetic process.” IL-11 can be expressed in many kinds of cells, even in lung fibroblasts, alveolar, and airway epithelial cells (Elias et al., 1994a,b). In addition, overexpression of IL-11 can also lead to subepithelial fibrosis and promote the accumulation of myocytes and myofibroblasts (Tang et al., 1998). As a member of IL gene family, IL-1a can be releases from damaged epithelial cells and trigger inflammatory responses in lung fibroblasts (Suwara et al., 2014). Our results were consistent with those of the other studies, indicating that fibrosis and immune response interacted. Interestingly, plenty of pax genes were enriched in GO terms, which were related to nervous system and epithelial cell development. pax8 is reported to play pivotal roles in renal regeneration upon acute injury (Narlis et al., 2007) and survival and proliferation of epithelial cells (Di Palma et al., 2013). pax8 is overexpressed in idiopathic pulmonary fibrosis (IPF) fibroblasts, which suggests that it may be a regulator in promoting the growth, survival, and proliferation of fibroblasts (Sheu et al., 2019). All these results are in line with the wound repair phase model: (1) injury, (2) inflammation, and (3) repair. It indicates that NGF, IL-1a, IL-11, and pax8 couple with each other and serve a role in the processes of injury, inflammation, and repair.

LncRNA is a novel class of mRNA-like transcripts with sizes varying from 200 to 100 kb and plays functions in regulating various BPs (Caley et al., 2010; Nagano and Fraser, 2011; Lee, 2012). In our study, many lncRNA-related pulmonary fibrosis were identified in GO and KEGG enrichment. It was shown that lncRNA MSTRG.23831.1 targeted in FBN1 and regulated TGF-β signal pathway. TGF-β occupies a set of functions including cellular differentiation, proliferation, cancerogenesis, and apoptosis. As a multifunctional cytokines, TGF-β plays a central role in wound healing, induces epithelial–mesenchymal transition (EMT), and leads to pulmonary fibrosis (Willis and Borok, 2007; Fernandez and Eickelberg, 2012). Apart from these, another two lncRNA–mRNA pairs (lncRNA MSTRG.24730.1-CASS4 and lncRNA MSTRG.38436.6-GDPD2) were significantly enriched into GO term “actin filament reorganization.” Actin cytoskeleton promotes myofibroblast differentiation and matrix remodeling during fibrogenesis (Sandbo and Dulin, 2011). Under stimulation of TGF-ββ1, ultrastructure of fibroblasts is altered with an increase in cytoskeletal stress fiber formation (Desmoulière et al., 1993; Vaughan et al., 2000). According to the target prediction results, lncRNA MSTRG.19006.1 may cis-regulate Adamts12 and then participate in the GO term “negative regulation of cellular response to VEGF stimulus.” VEGF is a key regulator of angiogenesis, which has been implicated in the pathogenesis of fibrotic lung disease and can also be regulated by TGF-β (Ward and Hunninghake, 1998; Magro et al., 2003; Ando et al., 2010). Taken together, we can find that TGF-β serves as a central factor in the regulating process of pulmonary fibrosis including cytoskeleton system and angiogenesis. KEGG enrichment analysis also supported the conclusion, such as “Notch signaling pathway” and “regulation of actin cytoskeleton.” Notch can activate the TGF-β/Smad signal and mediate the induction of SMA gene expression and myofibroblast differentiation in alveolar epithelial cells (Aoyagi-Ikeda et al., 2011). Co-expression network analyses show that Lrrk and RF00100 were the hub ncRNAs in pulmonary fibrosis. Lrrk could code leucine-rich repeat kinase, and it was related with Parkinson’s disease. In addition, it had the function of autophagy by autophagy–lysosomal pathway (Giaime et al., 2017). This might connect with fibrosis. The specific mechanism should be further studied by gene knocking out.

In this study, DE circRNAs were also identified. Four circRNAs (circRNA1863, circRNA3902, circRNA822, and circRNA178) participated in the GO term “negative regulation of epidermal growth factor receptor signaling pathway.” Seven circRNAs (circRNA1388, circRNA1765, circRNA243, circRNA2783, circRNA437, circRNA439, and circRNA988) regulated TGF-β signaling pathway. Seven circRNAs (circRNA1178, circRNA1355, circRNA1635, circRNA170, circRNA1765, circRNA3100, and circRNA822) played important roles in GO term “positive regulation of JNK cascade.” EGFR is overexpressed in fibrotic lung tissue, which suggests that EGFR signaling pathway is involved in epithelial regeneration in fibrotic lung diseases (Suzuki et al., 2003). All the results indicated that DE circRNAs did participate in plenty of aspects involved in the regulation of pulmonary fibrosis process.

The potential roles of DE miRNAs were investigated by GO and KEGG enrichment analyses of predicted target genes in this study. It was found that many miRNAs targeted in BCL gene family members such as bta-miR-339a, cpo-miR-424-5p, eca-mir-8969-p5, rno-miR-3590-3p, and rno-miR-19b-3p. It has been indicated that BCL-2 gene family members play a crucial role in the pathogenesis of inflammation, apoptosis, and fibrosis. Moreover, apoptosis serves as a critical role in wound repair and in the pulmonary epithelial injuries leading to fibrosis (Safaeian et al., 2014). DE miRNAs also participated in angiogenesis process. rno-miR-195-5p is targeted in the 3’ UTR of EREG, rno-miR-128-3p and rno-miR-27b-3p are targeted not only in the 3’ UTR of R−spondin3 but also in notch1, and rno-miR-672-3p is targeted in the 3’ UTR of HIF1a and angpt2. R−spondin3 is significantly expressed in blood-forming organs, and the deficiency of R−spondin3 would lead to lethal vessel remodeling defects. It could also promote vascular development through up-regulating VEGF by the activation Wnt signaling pathway. R−spondin3 keeps the balance between angiogenesis and hematopoiesis (Aoki et al., 2008; Kazanskaya et al., 2008). It was reported that HIF1a was highly expressed in bleomycin-induced mouse models of lung injury (Gross and Hunninghake, 2001; Tzouvelekis et al., 2007; Ueno et al., 2011; Malli et al., 2013). In addition, HIF1a even could up-regulate the adora2b receptor on alternatively activated macrophages and contribute to pulmonary fibrosis (Philip et al., 2017). Taken together, it indicated that DE miRNAs have participated in the process of pulmonary fibrosis by apoptosis, angiogenesis, and Wnt signaling pathway.

In this study, the expression profiles of lncRNAs, mRNAs, circRNAs, and miRNAs were detected by whole transcriptome sequencing in pulmonary fibrosis model and control lung tissues. After differential expression analysis, many mRNAs and ncRNAs were differentially expressed, and the GO and KEGG enrichment analyses allowed us to investigate the potential functions of these DE mRNAs and ncRNAs in the regulating process of pulmonary fibrosis. In addition, two co-expression networks (lncRNA–miRNA–mRNA and circRNA–miRNA–mRNA) were also constructed to understand the regulatory relations of these mRNAs and ncRNAs. It was speculated that pulmonary fibrosis was regulated by DE ceRNA related with apoptosis, angiogenesis, and immunology. Co-expression networks investigated that ncRNAs (Lrrk and RF00100) and miRNAs (miR-22-3p, miRNA-672-3p/5p, miR-20a-5p, miR-195-5p, let-7i-5p, miR-27b-3p, and miR-128-3p) were the hub RNAs. The results of this study provide a new insight and facilitate further studies into the genetic basis of pulmonary fibrosis and ceRNA mechanism of ncRNAs and mRNAs during the regulatory process of pulmonary fibrosis.

## Data Availability Statement

The datasets generated for this study can be found in NCBI GEO accession number of GSE153296 (https://www.ncbi.nlm.nih.gov/geo/query/acc.cgi?acc=GSE153296).

## Ethics Statement

The animal study was reviewed and approved by the Affiliated Hospital of Shandong University of Traditional Chinese Medicine.

## Author Contributions

WZ designed the experiment. XL and HL analyzed the transcriptome. XJ and XL wrote the manuscript. RH and XZ collected the samples. All authors contributed to the article and approved the submitted version.

## Conflict of Interest

The authors declare that the research was conducted in the absence of any commercial or financial relationships that could be construed as a potential conflict of interest.

## Footnotes

1 http://www.bioinformatics.babraham.ac.uk/projects/fastqc/

2 http://rna.tbi.univie.ac.at/cgi-bin/RNAfold.cgi

## Supplementary Material

Supplementary Figure S1 GO enrichment analysis of DE ceRNAs. **(A)** GO enrichment analysis of DE mRNAs. **(B)** GO enrichment analysis enrichment of DE lncRNAs. **(C)** GO enrichment analysis enrichment.
