# Supplementary material for: Changing Expression Profiles of Messenger RNA, MicroRNA, Long Non-coding RNA, and Circular RNA Reveal the Key Regulators and Interaction Networks of Competing Endogenous RNA in Pulmonary Fibrosis
Source: Front Genet. 2020 Sep 24;11:558095. doi: 10.3389/fgene.2020.558095 (PMC7541945; doi:10.3389/fgene.2020.558095)
Supplement: Supplementary Figure S1 — GO enrichment analysis of DE ceRNAs. (A) GO enrichment analysis of DE mRNAs. (B) GO enrichment analysis enrichment of DE lncRNAs. (C) GO enrichment analysis enrichment. [file Data_Sheet_1.zip › Table 1.DOCX]

**Table S1** List of primer sequences used in the study.

| Primers | Sequence (5′-3′) | Tm (°C) | Usage |
| --- | --- | --- | --- |
| Tnfrsf17-Fw  Tnfrsf17-Rv  IL-11-Fw | GTGACTATGGCAAGGGTATG  CTTCACATCCCACCAATTAGA  GCTCACCTGTGGCTTATTT | 60  60  60 | qRT-PCR  qRT-PCR  qRT-PCR |
| IL-11-Rv | GTGAGGAAGACACTGTGAATAG | 60 | qRT-PCR |
| Rasd1-Fw | GCTCACCTGTGGCTTATTT | 60 | qRT-PCR |
| Rasd1-Rv | GTGAGGAAGACACTGTGAATAG | 60 | qRT-PCR |
| IL-1a-Fw | GGCCATAGCCCATGATTT | 60 | qRT-PCR |
| IL-1a-Rv | CCTGCTTGACGATCCTTATC | 60 | qRT-PCR |
| Lair1-Fw | GCCACCAAATGCTTCTCT | 60 | qRT-PCR |
| Lair1-Rv | GTCTTTCCAAGGGCTGATAC | 60 | qRT-PCR |
| miR-676 | CCGTCCTGAGCTTGTCGAGCT | 60 | qRT-PCR |
| miR-2424 | ACAGATCTTTGGTAATCTGATGGCT | 60 | qRT-PCR |
| miR-1247-5p | ACCCGTCCCGTTCGTCCCCGGA | 60 | qRT-PCR |
| miR-3590-3p | TAGCACAATGTGAAAAGAGCTCT | 60 | qRT-PCR |
| miR-9995-3p | ATCTCGGTGGAACCTCCA | 60 | qRT-PCR |
| MSTRG.199-Fw | TGGTGCTTGTGCCTTTAC | 60 | qRT-PCR |
| MSTRG.199-Rv | CTCACCCAACGGAAGGA | 60 | qRT-PCR |
| MSTRG.11560-Fw  MSTRG.11560-Rv  MSTRG.11559-Fw  MSTRG.11559-Rv  MSTRG.30244-Fw  MSTRG.30244-Rv  MSTRG.15160-Fw  MSTRG.15160-Rv | CTCTCGTACTGAGCAGGATTA  GAACGTGAGCTGGGTTTAG  CTCCAGAGGTCCTGAGTTT  CTTCAGACACACCAGAAGAAG  TTAGAGGCGTTCAGTCATAATC  CCGCAGGTTCAGACATTT  GTGCCGCTTTACCCATATT  CCTCAACACCTGTGAGTTTC | 60  60  60  60  60  60  60  60 | qRT-PCR  qRT-PCR  qRT-PCR  qRT-PCR  qRT-PCR  qRT-PCR  qRT-PCR  qRT-PCR |
