# Supplementary figures and images for: Changing Expression Profiles of Messenger RNA, MicroRNA, Long Non-coding RNA, and Circular RNA Reveal the Key Regulators and Interaction Networks of Competing Endogenous RNA in Pulmonary Fibrosis
Source: Front Genet. 2020 Sep 24;11:558095. doi: 10.3389/fgene.2020.558095 (PMC7541945; doi:10.3389/fgene.2020.558095)

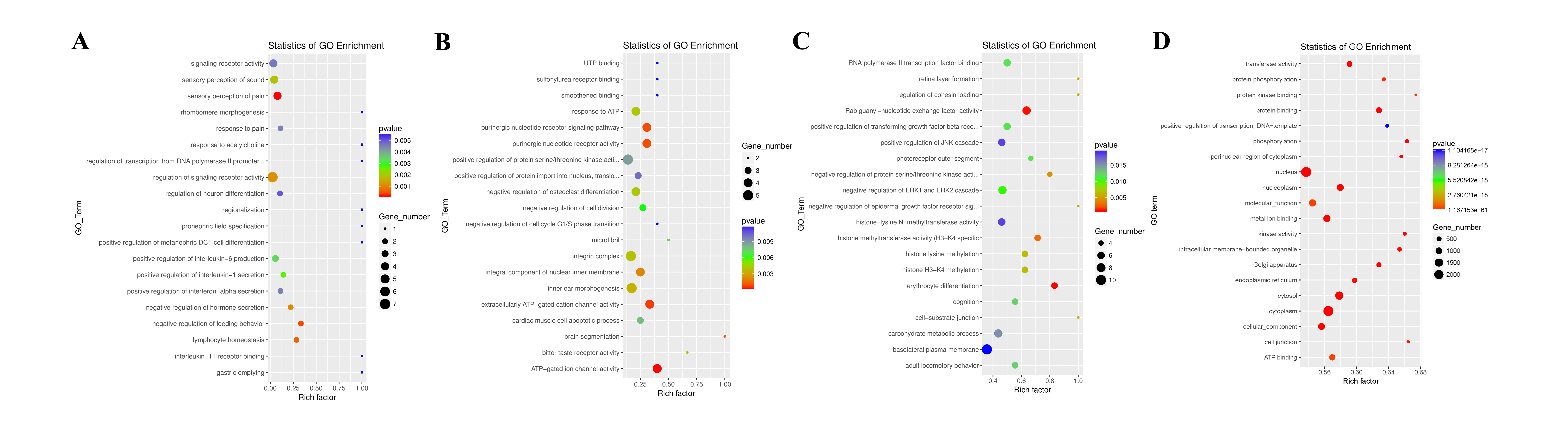

Supplement: Supplementary file 2 [file Image_1.TIF]
